# Investigation of Rutting and Aging Performance of Gap-Graded Rubberized Asphalt Mixtures

**DOI:** 10.3390/ma18102263

**Published:** 2025-05-13

**Authors:** Marek Pszczola, Bohdan Dolzycki

**Affiliations:** Faculty of Civil and Environmental Engineering, Gdansk University of Technology, 80-233 Gdansk, Poland; bohdan.dolzycki@pg.edu.pl

**Keywords:** gap-graded asphalt mixtures, crumb rubber modification, polymer–rubber-modified bitumen, rutting and aging resistance

## Abstract

Gap-graded asphalt mixtures like stone mastic asphalt (SMA), porous asphalt (PA), and asphalt mixtures for very thin layers (fr. Béton Bitumineuse Très Mince—BBTM) are usually made with the use of SBS (styrene-butadiene-styrene) polymer-modified bitumen. This is a binder that allows one to achieve the required parameters, but at the same time, its use increases the costs of making pavement layers. An alternative to polymer-modified bitumen (SBS) is rubber-modified bitumen. The research presented in this publication includes an assessment of the resistance to permanent deformation and susceptibility to aging of SMA and porous asphalt (PA) mixtures containing both SBS polymer-modified bitumen and rubber-modified bitumen, where the modification process was carried out directly in the refinery. The laboratory tests of resistance to deformation were assessed based on the rutting test and on the assessment of the dynamic modulus (SPT). The changes in the tested asphalt mixtures after aging in laboratory conditions were assessed based on the changes in the stiffness modulus (IT-CY) and the changes in the indirect tensile strength (ITS) after the short-term and long-term aging processes. The presented research results clearly show that the use of rubber-modified bitumen produced in industrial conditions (i.e., in a refinery) allows one to obtain gap-graded mixtures that are as resistant to permanent deformation as mixtures containing SBS polymer-modified bitumen. Similar conclusions resulted from the study of susceptibility to aging. Changes after aging for both types of asphalt mixtures were at a similar level. The presented results clearly indicate that, in the case of gap-graded mixtures such as SMA- and PA-type mixtures, they meet the rutting and aging expectations when either expensive modified bitumen or a cheaper, more environmentally friendly alternative (rubber-modified bitumen) is used.

## 1. Introduction

Gap-graded asphalt mixtures, such as stone mastic asphalt (SMA), porous asphalt (PA), and asphalt mixtures for very thin layers (Béton Bitumineuse Très Mince—BBTM), have gained widespread use in pavement construction, especially in milder-climate regions, due to their superior durability, resistance to rutting, and ability to improve skid resistance and noise reduction [1,2]. These mixtures, characterized by the absence of intermediate aggregate sizes, form a coarse aggregate skeleton with increased stone-on-stone contact, which significantly enhances rutting resistance and load-bearing capacity [3]. According to Yue et al. [4], in their study, gap-graded asphalt mixtures improved water damage tolerance, especially in hot climates, due to their thicker asphalt film and optimized aggregate structure. Xu et al. [5] showed that porous asphalt pavements significantly reduce noise, even during rain, by weakening the “pump suction” and “trumpet” effects that generate loud tire noises. This not only improves environmental conditions but also helps drivers stay more focused in wet-weather conditions. On the other hand, Xu et al. [5] highlighted the maintenance challenges in pavements made with use of gap-graded asphalt mixtures, where clogging of air voids over time diminishes permeability and noise reduction performance, necessitating regular surface treatments or cleaning.

Gap-graded asphalt mixtures typically incorporate polymer-modified bitumen (PmB), specifically styrene-butadiene-styrene (SBS)-modified bitumen, which enhances the mechanical performance of asphalt mixtures by improving their resistance to permanent deformation, fatigue cracking, and oxidative aging [6,7]. However, SBS-modified bitumen significantly increases material costs, raising concerns about the economic feasibility of large-scale road infrastructure projects [8].

An alternative to SBS-modified bitumen is polymer–rubber-modified bitumen (RMB), which is obtained by incorporating recycled rubber—often from waste tires—into the bitumen mixtures. This modification process promotes environmental sustainability by reducing tire waste while simultaneously improving the binder’s elasticity, thermal stability, and durability [9,10]. Studies have shown that polymer–rubber-modified bitumen can achieve performance levels comparable to SBS-modified bitumen, particularly in terms of aging resistance and rutting performance [11,12]. Bitumen modification with rubber can be carried out using two primary approaches: (i) the wet process, in which rubber particles are blended into bitumen at asphalt plants, and (ii) the industrial (refinery-based) process, where polymer–rubber-modified bitumen is produced under controlled conditions before transportation to the construction site [13]. The method of production in the refinery method, which, in the literature, is also called the “terminal blend” method, ensures better homogeneity, stability, and consistency in terms of the binder’s performance [14,15]. Swieczko-Zurek et al. [16] stated that, according to the mix of crumb rubber and SBS polymer, applied technology allows one to reduce the amount of added SBS polymer with respect to the standard polymer-modified bitumens. Also, the new material fulfills all requirements set for PmB bitumen according to the European standard for polymer-modified bitumen, EN 14023 [17], which is very important for the material acceptance process in road investment projects. Pszczola et al. [18] conducted research on the low-temperature properties of rubberized asphalt mixtures and proved that rubber–polymer-modified bitumen presented similar resistance to low-temperature action than SBS polymer-modified bitumen. Despite these advantages, a comprehensive evaluation of SBS-modified and rubber-modified bitumen in gap-graded mixtures such as SMA and PA mixtures in higher temperatures is still required.

The objective of this research was to assess the performance of SMA and PA mixtures containing both SBS polymer-modified bitumen and rubber-modified bitumen produced in industrial conditions. The key areas of evaluation included resistance to permanent deformation through rutting tests and dynamic modulus analysis, as well as susceptibility to aging through stiffness modulus (IT-CY) and indirect tensile strength (ITS) measurements following short-term and long-term aging. The results will provide valuable insights into the feasibility of rubber-modified bitumen as a cost-effective and environmentally sustainable alternative to SBS-modified bitumen in high-performance asphalt pavements.

## 2. Materials and Methods

### 2.1. Materials

Laboratory tests were conducted on two types of asphalt mixtures for the wearing course—stone matrix asphalt (SMA 8) and porous asphalt (PA 8). Aggregate skeleton was designed in compliance with Technical Guidelines [19] and based on European standards (EN 13108-5 [20] for SMA mixtures and EN 13108-7 [21] for PA mixtures). For each type of mixture, one optimum bitumen content was selected based on laboratory tests results for typical SBS-modified bitumen. The composition of the mixtures and types of bitumen used are presented in Table 1.

Four types of bitumen were selected for rutting and aging performance tests, including two types of rubber–polymer-modified bitumen—45/80-55 CR and 45/80-65 CR—and, as references, two types of SBS polymer-modified bitumen—45/80-55 and 45/80-65. The standard properties of the bitumens used in this research are shown in Table 2.

Before specimen compaction, every type of asphalt mixture was subjected to short-term aging according to standard specification AASHTO R30-02 [22].

**Table 2:** The properties of the bitumens used in the asphalt mixtures according to European standard EN 14023.

Property:	Type of Bitumen			
	45/80-55 PmB	45/80-55 CR	45/80-65 PmB	45/80-65 CR
Penetration in 25 °C, 0.1 mm, acc. EN 1426 [23]	43	53	52	45
R&B Temperature, °C, acc. EN 1427 [24]	60	55	72	76
Dynamic viscosity, Pa·s, acc. EN 12596 [25]				
90 °C	35.321	19.058	43.728	81.833
135 °C	1.225	0.859	1.813	1.947
160 °C	0.373	0.303	0.596	0.563

### 2.2. Methods

#### 2.2.1. Resistance to Rutting Test Methods

Samples for laboratory testing were compacted in steel molds with internal dimensions of 300 × 300 × 40 mm at a temperature of 145 ± 5 °C until a compaction level of 98–100% was achieved. Samples were prepared and compacted successively in such a way so as to obtain the same time between compaction and testing of samples, ranging from 6 to 8 days. Immediately before testing, the samples were conditioned at a test temperature of 60 °C for at least 4 h but no longer than 18 h.

The rutting resistance of the asphalt mixtures was tested based on the European standard EN 12697-22 [26]. All values were assessed as the average value of two test results. The resistance of the asphalt mixtures to rutting was assessed based on the value of rut depth, RD_AIR_, formed as a result of repeated passes of a loaded wheel at a constant temperature. During the test, the rut depth measured over the rut length was recorded.

#### 2.2.2. Dynamic Modulus Test Method in AMPT/SPT

**Figure 1:**
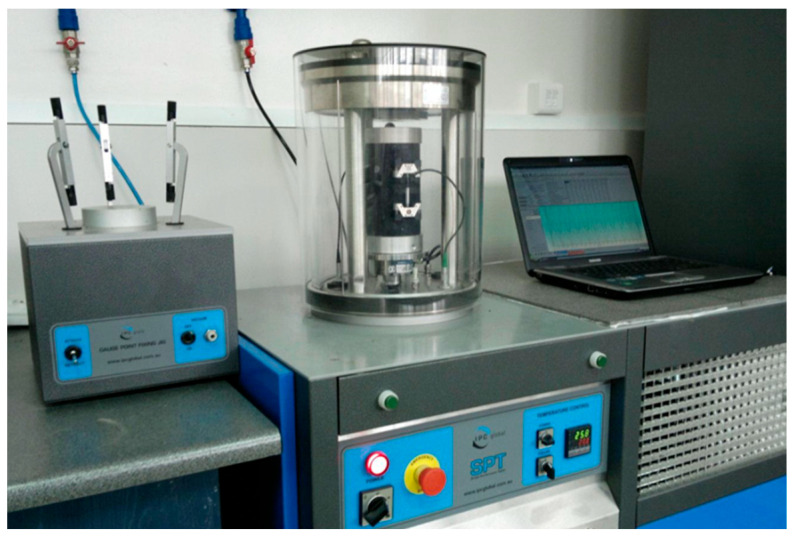
Image of the Asphalt Mixture Performance Tester/Simple Performance Test (AMPT/SPT) equipment IPC Global, Boronia, Australia.

The test was performed on cylindrical samples with a diameter of 100 mm and a height of 150 mm, which were compacted in a gyratory press at a temperature of 145 ± 5 °C until the compaction level was within the range of 98–100%. Before the test, the upper and lower surfaces of the samples were precisely cut to obtain two flat and parallel planes. The dynamic modulus test was performed based on the proposal of the American guidelines NCHRP 9-29: PP 02 [27]. This test is an improved and simplified version of the dynamic modulus test based on the AASHTO TP62 standard [28]. The dynamic modulus test consisted of axial loading of a cylindrical sample with a vertical sinusoidal force at specified frequencies and measuring deformations using three LVDT sensors mounted equidistantly on the side of the cylindrical sample at a spacing of 120°. Figure 1 shows an image of the device and the method of mounting the sample in the device.

The values measured in the test were the dynamic modulus and phase angle. The load frequencies that were set by the device were as follows: 25 Hz, 20 Hz, 10 Hz, 5 Hz, 2 Hz, 1 Hz, 0.5 Hz, 0.2 Hz, 0.1 Hz, and 0.01 Hz. The test procedure consisted of placing the sample in a chamber in which a constant test temperature was maintained. Then, the sample was loaded axially. The measurement of the dynamic modulus and phase angle was performed at 3 temperatures (+4 °C, +20 °C, and +40 °C) in the full frequency range from 25 Hz to 0.01 Hz. The test at a frequency of 0.01 Hz was performed only for the highest temperature. For each temperature, 3 cylindrical specimens were tested. The dynamic modulus tests were performed to obtain the characteristics of asphalt mixtures in the form of master curves. To determine the equation of the master curve for the dynamic modulus, we used the formula provided in the NCHRP 9-29 guidelines: PP 02 [27]:

(1) $$log\left|E^{*}\right|=\delta +\frac{\left(Max-\delta \right)}{1+e^{\beta +\gamma \left\{\log\omega +\frac{∆E_{a}}{19.14714}\left[\left(\frac{1}{T}\right)-\left(\frac{1}{T_{r}}\right)\right]\right\}}}$$

where

|E*|—dynamic stiffness modulus, psi (1 MPa = 145.0377 psi);

*Max*—maximum limiting modulus of the leading curve, psi;

*T_r_*—reference temperature, °K;

*T*—test temperature, °K;

Δ*E_a_*—activation energy (treated as a curve fitting parameter);

*δ, β, γ*—curve fitting parameters.

To determine the reduced frequency when determining the leading curve, the Arrhenius formula was used:

(2) $$\log f_{r}=\log f+\frac{∆E_{a}}{19.14714}\left(\frac{1}{T}-\frac{1}{T_{r}}\right)$$

where

*f_r_*—reduced frequency at reference temperature, Hz;

*f*—load frequency at test temperature, Hz.

**Figure 2:**
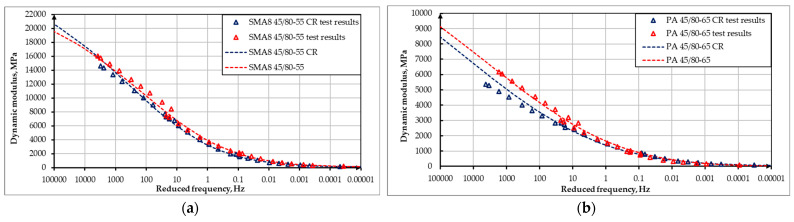
Master curves developed for tested asphalt mixtures, where black color dots represent results of mixtures incorporating polymer–rubber-modified bitumen and red color dots represent results of mixtures incorporating SBS polymer-modified bitumen: (**a**) SMA mixture; (**b**) PA mixture.

The reference temperature was set to 20 °C. Master curves developed for the tested asphalt mixtures are presented in Figure 2.

#### 2.2.3. Resistance to Aging Test Methods

The tests on the resistance of the analyzed asphalt mixtures to aging were carried out in accordance with the AASHTO R 30-02 procedure [22]. This procedure specifies the principles for preparing, conditioning, and testing the specimens subjected to aging:

- Short-term aging—Performed on a loose mixture, this simulates the aging of the mineral–asphalt mix during production and transport;
- Long-term aging—Performed on a compacted mixture, this simulates the operational aging of the mineral–asphalt mix in the layer after compaction.

During aging, the asphalt mixture stiffens. Long-term aging can cause it to become brittle and susceptible to cracking. Aging causes an increase in indirect tensile strength in higher summer temperatures and a decrease in low winter temperatures. The aim of this study was to compare changes in both types of asphalt mixes, differing in the type of asphalt, after the aging process.

**Figure 3:**
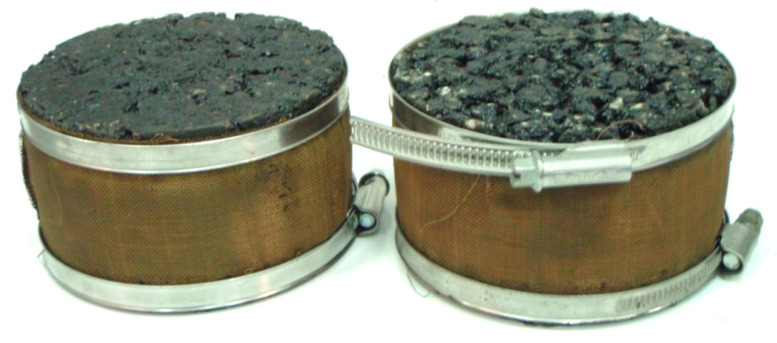
Image of specimens prepared for long-term aging testing.

The test was performed on cylindrical specimens with a diameter of 100 mm and a height of 63 mm, compacted by tamping (2 × 50 blow Marshall hammer) at a temperature of 145 ± 5 °C. Specimens with a diameter of D = 101 mm and a height of H = 63.5 mm were formed. The tests included unaged specimens (reference), specimens after short-term aging, and specimens after long-term aging. Each specimen group was conditioned as follows:

- Unaged specimens (reference), i.e., specimens not subjected to short-term and long-term aging—After the asphalt mixture was mixed and the mixture reached a temperature of 145 ± 5 °C, the specimens were compacted;
- Specimens after short-term aging—The asphalt mixture was mixed in a mechanical mixer at a temperature of about 145 ± 5 °C, and then the loose mixture was subjected to a short-term aging process. The mixture was spread in sheet metal forms so that the layer thickness was from 2.5 to 5.0 cm. The molds with the mixture were stored in an oven with forced air flow at a temperature of 135 ± 3 °C for 4 h ± 5 min. The mixture was cyclically mixed every hour. Then, the mixture was heated to a temperature of 145 ± 5 °C and the specimens were compacted;
- Specimens after long-term aging—Long-term aging involved storing the formed and compacted specimens after short-term aging in an oven with air flow for 5 days (120 ± 0.5 h) at a temperature of 85 ± 3 °C. The specimens were wrapped in a fine copper mesh to protect against possible deformations. An image of the samples prepared for long-term aging is shown in Figure 3.

The assessment of the aging of asphalt mixtures consisted of comparing the test results of unaged specimens (reference) with the test results of specimens subjected to aging. The adopted aging measures were as follows:

- Change in the stiffness modulus;
- Change in the indirect tensile strength.

The tests of the stiffness modulus and indirect tensile strength were performed at a temperature of 25 °C, after a minimum of 4 h of conditioning. Each specimen was tested twice:

- For the first time, they were tested by performing a non-destructive test of the stiffness modulus following an indirect tensile scheme (IT-CY);
- for the second time, they were tested by performing a destructive test of the indirect tensile strength.

The stiffness modulus test was performed following an indirect tensile scheme (IT-CY) in accordance with Annex C of the EN 12697-26 standard [29]. The load increase time was 120 ± 4 ms, and the duration of one cycle was 3 s. The test was carried out via a controlled deformation test with a horizontal deformation of 5 μm. The test result is the average value of the stiffness modulus measured in two perpendicular planes. The indirect tensile strength tests were performed on the same samples on which the stiffness modulus test was previously performed using the indirect tensile method. This test was performed via a press with a constant displacement rate of 50 mm/min. The load was transferred through spacers that were 12 mm wide and with a radius of curvature of 50.5 mm. The test determined the destructive force of the samples, and on this basis, the destructive stresses, i.e., the indirect tensile strength, were determined. The indirect tensile strength test was carried out at a temperature of +25 °C. In each of the two presented test methods, 3 specimens were tested for each asphalt mixture.

## 3. Results and Discussion

### 3.1. Test Results for Resistance to Permanent Deformation

**Figure 4:**
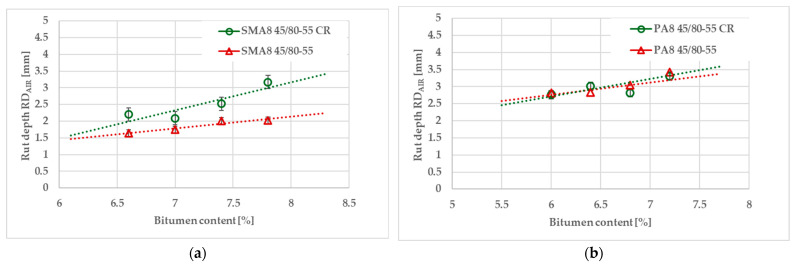
Results of rut depth RD_AIR_ obtained in the rutting resistance test according to EN 12697-22: (**a**) SMA mixture; (**b**) PA mixture.

The rutting test was carried out according to the methodology described in Section 2.2.1. The results regarding rutting resistance, defined as rut depth values, for the SMA and PA mixtures are presented in Figure 4. The resistance to permanent deformation test results are presented for several bitumen contents in order to simultaneously assess the sensitivity of the tested open graded asphalt mixtures to changes in the bitumen content according to possible deviations during the production of mixtures in the plant.

In the case of the PA8 mixture, regardless of the criterion analyzed, both modified bitumens, with and without rubber, show similar values. Both PA8 mixtures behave in a similar way. However, in the case of the SMA8 asphalt mixture, the rut resistance obtained only for the SBS-modified bitumen sample is slightly better compared to the SMA mixture containing rubber–polymer-modified bitumen. The rubberized SMA mixture can also be more susceptible to bitumen content variation during production.

### 3.2. Test Results for Dynamic Modulus in AMPT/SPT Equipment

**Figure 5:**
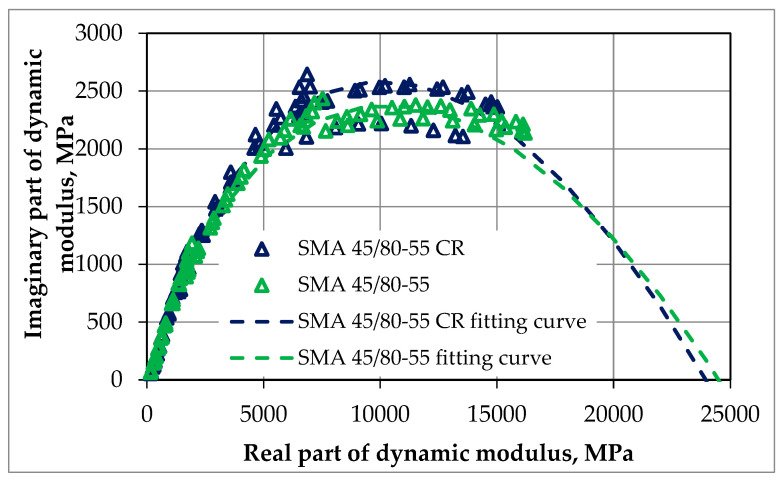
The results of our dynamic modulus testing of the SMA mixtures according to guidelines, NCHRP 9-29 [27]; the results are presented via a Cole–Cole diagram.

**Figure 6:**
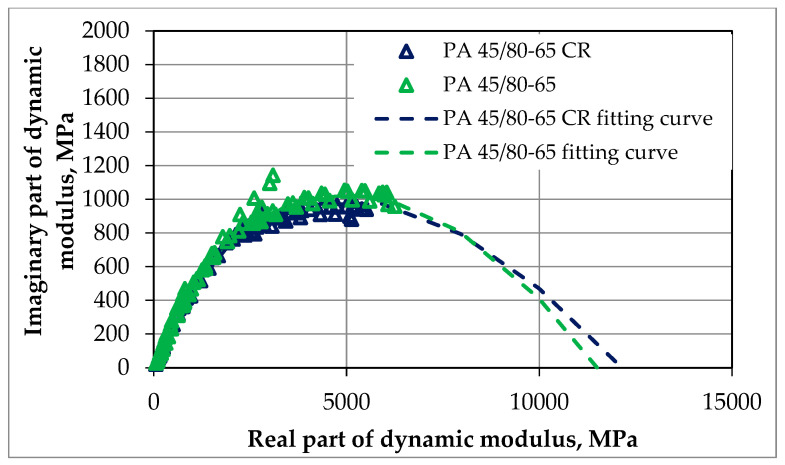
The results of our dynamic modulus testing of the PA mixtures according to guidelines, NCHRP 9-29 [27]; the results are presented via a Cole–Cole diagram.

**Figure 7:**
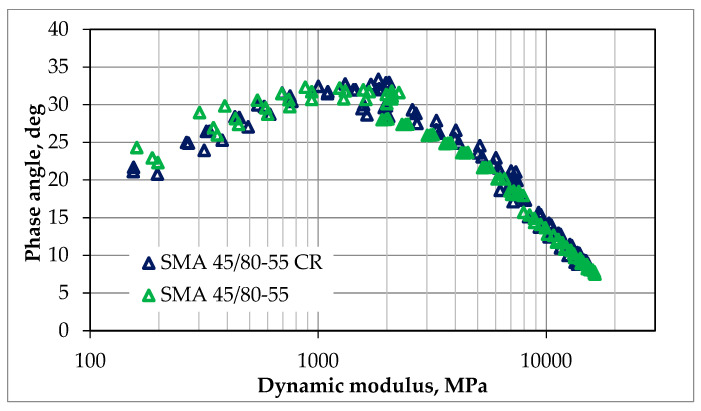
The results of our dynamic modulus testing of the SMA mixtures according to guidelines, NCHRP 9-29 [27]; the results are presented as Black curves.

**Figure 8:**
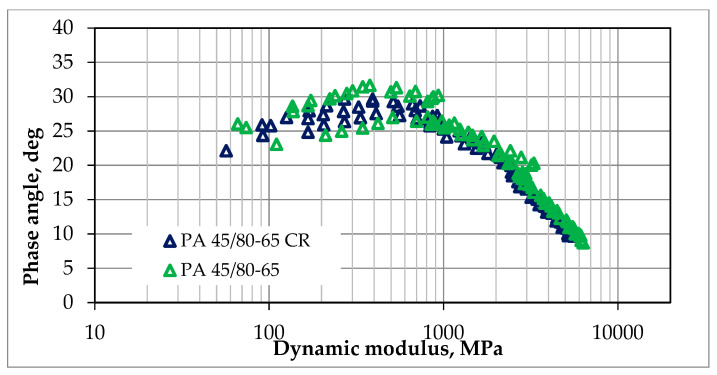
The results of our dynamic modulus testing of the PA mixtures according to guidelines, NCHRP 9-29 [27]; the results are presented as Black curves.

The dynamic modulus test was conducted according to the methodology described in Section 2.2.2. The resistance to deformation in this test was assessed by analyzing the obtained results of the master curves and comparing them. The tests were conducted for each of the mixtures for one optimal value of bitumen content, which was 7.0% for SMA 8 and 6.5% for PA8, respectively. The results of dynamic tests can also be presented in an unprocessed form, e.g., in the form of graphs of the following dependencies:

- The imaginary part of the dynamic modulus as a function of the real part of the dynamic modulus (Cole–Cole diagram)—as shown in Figure 5 and Figure 6;
- The phase angle as a function of the dynamic modulus (Black’s curve)—as shown in Figure 7 and Figure 8.

These graphs allow for the detection of nonlinearity in the behavior of asphalt mixtures and a comparison of the rheological properties of mixtures by directly presenting the results of the dynamic modulus test (without transposing the test results, as is the case when creating leading curves).

The Cole–Cole diagrams and Black curves presented do not show any significant differences in the behavior of mixtures made using SBS polymer-modified bitumen and rubber–polymer-modified bitumen (RMB).

### 3.3. Test Results and Analysis of the Resistance to Aging

The aging of the gap-graded asphalt mixtures was carried out according to the procedure described in Section 2.2.3. The resistance of the analyzed asphalt mixtures to aging was assessed by the following metrics:

- Index of stiffness modulus (ISM);
- Indirect tensile strength index (ITSI).

The index of stiffness modulus was defined as follows:

(3) $$ISM=\frac{mean\;value\;of\;stiffness\;modulus\;after\;aging}{mean\;value\;of\;stiffness\;modulus\;of\;reference\;specimens}\cdot 100\%$$

The indirect tensile strength index was defined as follows:

(4) $$ITSI=\frac{mean\;value\;of\;indirect\;tensile\;strength\;after\;aging}{mean\;value\;of\;indirect\;tensile\;strength\;of\;reference\;specimens}\cdot 100\%$$

The test results of the stiffness modulus and indirect tensile strength, as well as the indices calculated on the basis of the test results, are presented in Table 3.

Having conducted an analysis of the resistance of gap-graded asphalt mixtures to aging, we can make the following conclusions:

- In the case of the SMA mixture, the comparison of stiffness modulus indices shows that the use of rubber to modify bitumen did not affect either short-term or long-term aging;
- In the case of the porous asphalt (PA) mixture, the use of rubber to modify the bitumen significantly reduces the value of the index of stiffness modulus (ISM). The high values of the stiffness modulus obtained for the porous asphalt mixture in comparison with the SMA mixture are noteworthy. This may indicate that the PA mixture has greater susceptibility to aging, especially long-term aging;
- In the case of the SMA mixture, the comparison of indirect tensile strength indices shows that the use of rubber to modify bitumen has a beneficial effect on both short-term and long-term aging;
- In the case of the porous asphalt (PA) mixture, the addition of rubber to modified bitumen did not significantly affect the value of the indirect tensile strength index (ITSI). The values obtained regarding the indirect tensile strength indices for the porous asphalt (PA) mixture are not as high as those for the stiffness modulus indices;
- The tests validated our expectation that, with aging, the stiffness of the materials will increase, regardless of the bitumen used, especially in mixtures with higher void content values (PA 8). On the other hand, changes in the indirect tensile strength showed that, during aging, the material does not become more brittle and therefore more susceptible to cracking, regardless of the type of the mixture tested and the type of bitumen used.

## 4. Conclusions

The presented test results regarding the rutting and aging performance of gap-graded asphalt mixtures SMA8 and PA8 with SBS polymer- and CR-modified bitumen allow us to form the following conclusions:

1. Based on the rutting test, it can be concluded that polymer-modified bitumen supplemented with rubber allows for the design of asphalt mixtures that are resistant to permanent deformations and do not differ substantially from the same mixtures containing bitumen without the addition of rubber.
2. Dynamic moduli and phase angles show a less elastic character of mixtures made using rubber–polymer-modified bitumens (lower dynamic moduli and higher phase angles at temperatures from 4 °C to 40 °C), but the differences were not significant.
3. The aging tests showed that, with aging, the stiffness of the materials increases irrespective of the type of bitumen used, especially in the more open asphalt mixture (PA 8). The increase in stiffness after aging can be more significant depending on the gap-graded asphalt mixtures’ resistance to low-temperature cracking.

The research presented in this paper confirmed literature studies stating that the addition of rubber to SBS polymer bitumen does not significantly impair the properties of gap-graded asphalt mixtures in higher, summer temperatures. The PA- or SMA-type mixes made with rubber–polymer-modified bitumen behave in a similar way to those incorporating bitumen with SBS polymer modification. Laboratory tests obviously need to be verified in the field, but the results clearly show that it is possible to use cheaper rubber–polymer blended bitumens instead of more expensive SBS polymer-modified bitumens without degrading asphalt mixtures’ resistance to permanent deformation.

## Figures and Tables

**Table 1:** Composition of tested asphalt mixtures.

	Type of Asphalt Mixture			
Property	SMA8	SMA8 CR	PA8	PA8 CR
Passes, mm				
11.2	100.0		100.0	
8	94.2		91.2	
5.6	41.2		13.4	
2	25.6		6.7	
0.125	11.9		4.8	
0.625	9.7		4.1	
Type of aggregate	gneiss, granodiorite, and limestone		gneiss, granodiorite, and limestone	
Bitumen content, % *w*/*w*	7.0		6.5	
Type of bitumen	45/80-55 PmB	45/80-55 CR	45/80-65 PmB	45/80-65 CR

**Table 3:** The results of our aging assessment of open-graded asphalt mixtures.

Parameter Tested:	Open-Graded Asphalt Mixture			
	SMA8 45/80-55	SMA8 45/80-55 CR	PA8 45/80-65	PA8 45/80-65 CR
Stiffness Modulus at 25 °C, MPa				
Reference specimens	2270	1607	641	1103
After short-term aging, MPa	2937	2075	1023	1573
After long-term aging, MPa	3150	2465	1335	2088
Index of stiffness modulus, ISM				
ISM—after short-term aging, %	129	129	159	143
ISM—after long-term aging, %	138	153	208	189
Indirect tensile strength at 25 °C, MPa				
Reference specimens	1.08	1.01	0.43	0.53
After short-term aging, MPa	1.33	1.04	0.57	0.69
After long-term aging, MPa	1.24	1.09	0.57	0.74
Indirect tensile strength index, ITSI				
ITSI—after short term aging, %	123	103	132	130
ITSI—after long-term aging, %	115	108	132	139

## Data Availability

The original contributions presented in this study are included in the article. Further inquiries can be directed to the corresponding author.

## Abbreviations

The following abbreviations are used in this manuscript:

GGAM	Gap-graded asphalt mixture
SMA	Stone mastic asphalt
PA	Porous asphalt
BBTM	Béton Bitumineuse Très Mince
SBS	Styrene-butadiene-styrene
PMB	Polymer-modified bitumen
RMB	Rubber-modified bitumen
AMPT/SPT	Asphalt Mixture Performance Tester/Simple Performance Tester
ITS	Indirect tensile strength
CR	Crumb rubber
ISM	Index of stiffness modulus
ITSI	Indirect tensile strength index
